# Two exfoliation approaches for organic layered compounds: hydrophilic and hydrophobic polydiacetylene nanosheets†
†Electronic supplementary information (ESI) available: Experimental procedure, UV-Vis spectra, FT-IR spectra, XRD profiles and AFM images. See DOI: 10.1039/c6sc03350d


**DOI:** 10.1039/c6sc03350d

**Published:** 2016-08-30

**Authors:** Yukiko Ishijima, Mamoru Okaniwa, Yuya Oaki, Hiroaki Imai

**Affiliations:** a Department of Applied Chemistry , Faculty of Science and Technology , Keio University , 3-14-1 Hiyoshi, Kohoku-ku , Yokohama 223-8522 , Japan . Email: oakiyuya@applc.keio.ac.jp

## Abstract

Nanosheets have attracted much interest because of their characteristic properties originating from anisotropic and flexible structures. Inorganic nanosheets are synthesized from precursor layered compounds through exfoliation in a liquid phase. In contrast, a versatile exfoliation approach has not been fully studied for organic layered compounds. Here we report two exfoliation approaches for organic layered compounds. Hydrophilic and hydrophobic polydiacetylene (PDA) nanosheets, around 5 nm in thickness, are obtained through exfoliation of the layered precursor in aqueous and nonpolar organic media, respectively. The intercalation of ions and molecules in the interlayer space facilitates swelling and exfoliation. The resultant PDA nanosheets showed characteristic photochemical properties originating from the flexible structure. The exfoliation approach can be applied to a variety of organic layered compounds for the generation of designed nanosheets.

## Abstract 2

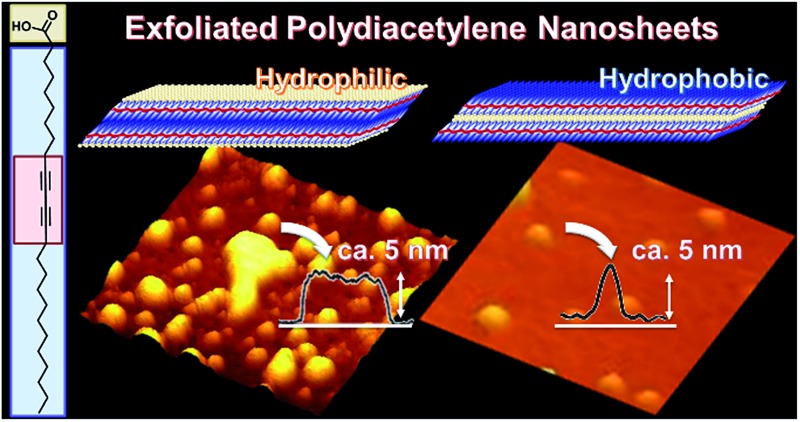
Two versatile exfoliation approaches generate both hydrophilic and hydrophobic nanosheets from an organic layered compound.

## Introduction

**Fig. 1:**
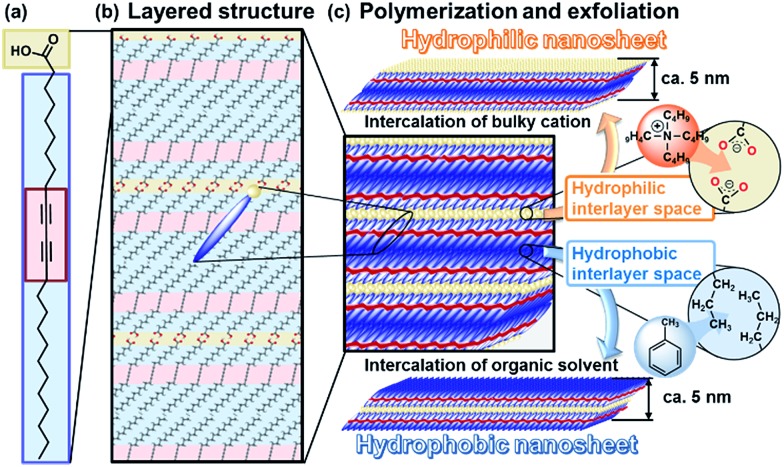
Schematic illustration of the layered PDA and its exfoliation into hydrophilic and hydrophobic nanosheets. (a) Molecular structure of the PCDA monomer. (b) Layered crystal structure of PCDA with the hydrophilic (gold color) and hydrophobic (blue color) interlayer space. (c) Topochemical polymerization and subsequent exfoliation into hydrophilic and hydrophobic PDA nanosheets consisting of a bilayered molecular arrangement approximately 5 nm in thickness.

Nanosheets, including monolayered and few-layered structures, have been studied in a variety of inorganic and organic compounds.^1–7^ Nanosheets show emerging properties originating from their characteristic structures,^8–11^ such as high specific surface area, flexible nature, and two-dimensional anisotropy. In general, intercalation facilitates exfoliation of the precursor layered compounds in a liquid phase through the cleavage of interactions between the layers. Exfoliation methods have been reported for inorganic layered compounds, such as clays, layered double hydroxides, transition metal chalcogenides, and transition metal oxides.^1,2,5–7,11–15^ In contrast, exfoliation methods have not been fully studied for organic layered compounds. If rational exfoliation approaches are developed for organic layered compounds, a variety of organic nanosheets can be obtained with designed molecular structures and properties. The present study shows two exfoliation approaches for the synthesis of hydrophilic and hydrophobic organic nanosheets from layered polydiacetylene (PDA) as a model of the layered precursor compound (Fig. 1).

Inorganic layered compounds with weak interactions between the layers, such as clays and transition metal chalcogenides, undergo exfoliation with dispersion in aqueous and polar organic media.^1,5,6^ In contrast, it is not easy to achieve delamination of inorganic layered compounds with strong interactions between the layers, such as metal oxides.

Two general exfoliation methods based on intercalation have been developed for syntheses of hydrophilic and hydrophobic nanosheets dispersed in aqueous and nonpolar organic media, respectively.^2,12,16–18^ Layered transition-metal oxides consist of a metal-oxide layer and interlayer ions. Intercalation of guest bulky ions and molecules in the interlayer space promotes swelling of the interlayer space.^2,12,16^ The hydrophilic and charged inorganic monolayers are exfoliated and dispersed in aqueous and polar organic media. Our group has developed another exfoliation method to obtain hydrophobic inorganic monolayers dispersible in nonpolar organic media.^18^ When the interlayer space of layered compounds is modified by hydrophobic organic molecules, such as long-chain alkyl ammonium ions, the intercalation and swelling proceed in nonpolar organic media. The surface-modified hydrophobic inorganic monolayers are exfoliated and dispersed in nonpolar organic media.^18^ However, these rational approaches cannot be effectively applied to the exfoliation of organic layered compounds because of structure differences. Our intention here is to develop versatile exfoliation approaches for organic layered compounds. In the present work, hydrophilic and hydrophobic organic nanosheets were obtained through exfoliation approaches inspired by those for inorganic layered compounds (Fig. 1).

Lamellar structures of organic molecules, such as Langmuir–Blodgett (LB) films and liquid crystals, are formed through self-assembly of the molecules.^19,20^ Two-dimensional (2D) polymers have attracted much attention as organic layered compounds and organic clays.^21^ In the 2D polymers, the layers consist of a planar network of self-assembled molecules with covalent and/or noncovalent bonds. The layers are stacked *via* van der Waals interactions and/or π–π stacking. The direct synthesis of organic nanosheets was achieved through the programed assembly of designed molecules.^22–24^ The exfoliation of organic layered compounds has been studied in a limited number of previous works.^25^ A review paper indicates that the exfoliation is not easily performed because of the strong stacking between the layers.^21*c*^ In the present work, two exfoliation approaches were studied for the exfoliation of organic layered compounds into hydrophilic and hydrophobic nanosheets through intercalation and swelling (Fig. 1). Here we focused on crystalline layered PDA as a model for the development of exfoliation schemes because the layered structure has hydrophilic and hydrophobic interlayer spaces for intercalation (Fig. 1c). The two exfoliation schemes for inorganic layered compounds can be applied to organic layered compounds with a hydrophilic and hydrophobic interlayer space. A diacetylene derivative of 10,12-pentacosadiynoic acid (PCDA) forms the layered crystal structure (Fig. 1a and b).^26,27^ The topochemical polymerization of PCDA provides PDA without a change of the layered structure (Fig. 1b and c). The intercalation of bulky cations in the hydrophilic interlayer space consisting of carboxy groups induces exfoliation into the hydrophilic PDA nanosheets in aqueous media. The hydrophobic PDA nanosheet is synthesized *via* the intercalation of nonpolar organic molecules in the hydrophobic interlayer space. These exfoliation approaches can be applied to a variety of organic layered compounds consisting of designed molecules with a hydrophilic and hydrophobic interlayer space.

## Results and discussion

### Precursor organic layered compounds

**Fig. 2:**
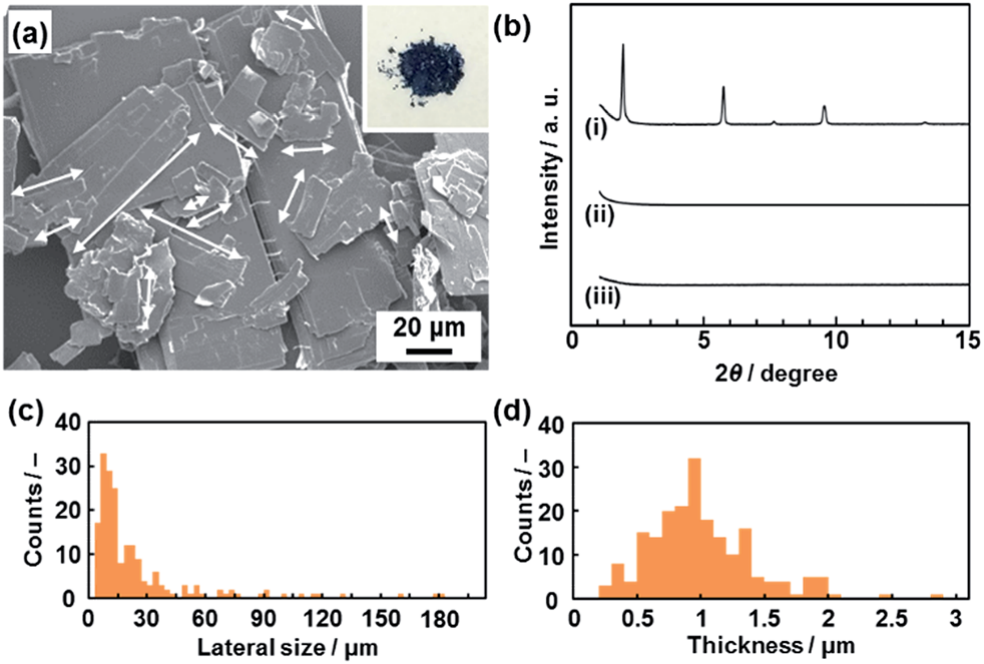
Precursor layered PDA crystals and their crystal structure changes after dispersion in aqueous and nonaqueous media. (a) SEM image of the PDA crystals and a photograph of the powder (the inset). (b) XRD patterns of the layered PDA before exfoliation (i), the hydrophilic PDA nanosheets collected after immersion in an aqueous solution containing TBAOH for 3 weeks (ii), and the hydrophobic PDA nanosheets after immersion in toluene for 3 weeks (iii). (c and d) Lateral size (c) and thickness (d) distribution of the precursor PDA crystals. The longitudinal length of platy primary units, as indicated by the white arrows in the panel (a), was measured as the lateral size.

A commercial powder of PCDA as the monomer crystal was polymerized with UV light and X-ray irradiation.^26^ The resultant PDA powder with a dark-blue color was used as the precursor layered compound (the inset of Fig. 2a). The detailed experimental methods are described in the ESI.†

The layered PDA showed aggregates of platy units 11.9 ± 9.0 μm in lateral size and 0.89 ± 0.38 μm in thickness (Fig. 2a, c and d). Diffraction peaks characteristic of the layered crystal structure were observed on the X-ray diffraction (XRD) pattern (profile (i) in Fig. 2b). The diffraction peaks at 2*θ* = 1.96°, 3.85°, 5.73°, 7.61°, 9.51°, and 13.3° are calculated to be spacings of 4.51 nm, 2.29 nm, 1.54 nm, 1.16 nm, 0.930 nm, and 0.664 nm, respectively, corresponding to the diffraction of *d*_0_/*n* (*n* = 1, 2, 3, 4, 5, 7) on the assumption of a layered crystal structure with an interlayer spacing of *d*_0_ = 4.51 nm (Fig. 1b).

### Exfoliation into hydrophilic PDA nanosheets

**Fig. 3:**
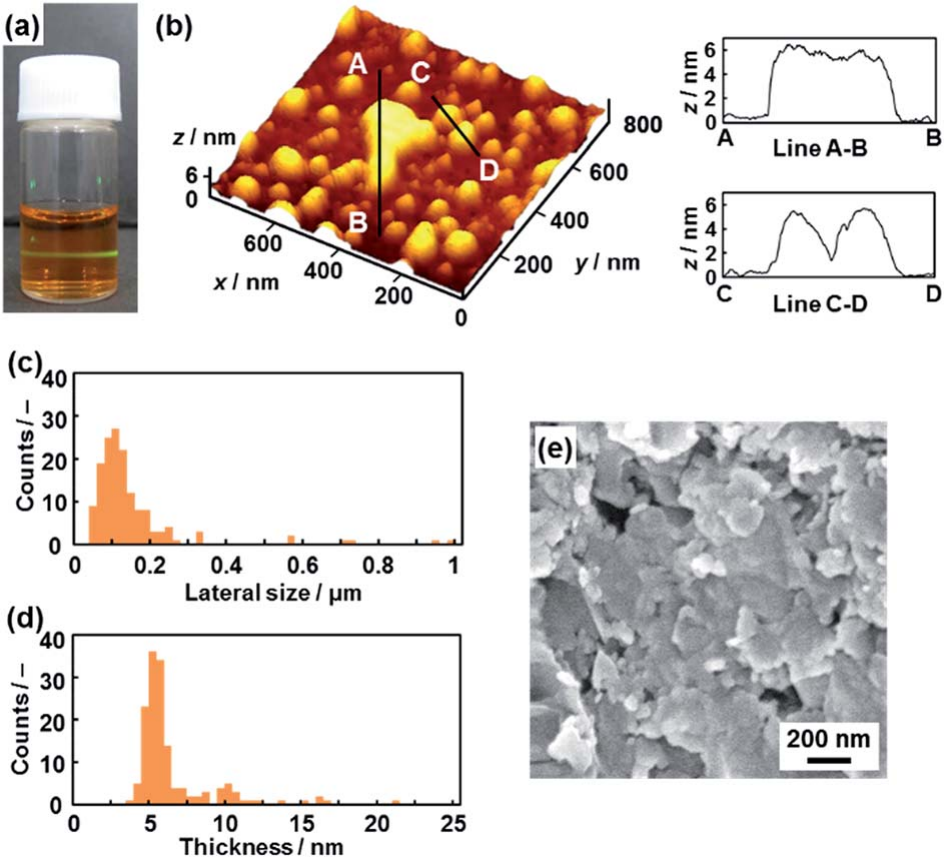
Hydrophilic PDA nanosheets. (a) Photograph of the aqueous dispersion liquid on irradiation with laser light. (b) AFM image of the hydrophilic nanosheets and their height profiles. (c and d) Distribution of the lateral size (c) and the height (d). (e) Scanning electron microscope images of the restacked hydrophilic PDA nanosheets collected with addition of zinc ions in the dispersion liquid. The additional AFM images are displayed in Fig. S2 in the ESI.†

The hydrophilic PDA nanosheets were obtained by dispersion of the layered PDA in an aqueous solution containing tetrabutylammonium hydroxide (TBAOH) (Fig. 3a–d). The molar ratio of TBAOH to the carboxy group in PDA (*R*_TBAOH/COOH_) was adjusted to 4.0. The color of the PDA powder changed from blue to red upon dispersion in the TBAOH aqueous solution. The color change is ascribed to shortening of the conjugation length with torsion of the diacetylene main chain by the application of external stimuli.^26,27^ Bulk precipitates were not observed in the dispersion liquid after 24 h. After 3 weeks, the remaining monomers and dissolved polymers are removed through separation by addition of ethyl acetate to the aqueous phase. A transparent dispersion liquid with an orange color was obtained (Fig. 3a). Light scattering was observed in the dispersion liquid on irradiation with laser light (Fig. 3a). The results imply that nanoscale objects are dispersed in the colloidal liquid. The color of the dispersion liquid gradually changed from red to orange within 3 weeks (Fig. S1 in the ESI†).

Nanosheets approximately 5 nm in thickness were observed in the atomic force microscope (AFM) images (Fig. 3b and S2 in the ESI†). The distribution of the lateral size and thickness is summarized in the histograms (Fig. 3c and d). Since the interlayer distance based on the interdigitated arrangement is *d*_0_ = 4.51 nm on the XRD pattern, the thickness of the nanosheet unit consisting of the bilayered molecular arrangement is estimated to be around 5 nm (Fig. 1c). Most of the nanosheets had a thickness around 5 nm even though a small amount of the thicker few-layer objects were present (Fig. 3d). The height distribution indicates that hydrophilic PDA nanosheets with different layer numbers were formed through exfoliation in aqueous media containing TBAOH (Fig. 1c). The average lateral size of the sheets was 102 ± 47 nm (Fig. 3c). The lateral size of the precursor layered structure was decreased from approximately 10 μm to 0.1 μm (Fig. 2c and 3c). The size reduction is ascribed to fracturing in the lateral direction during exfoliation. It is inferred that the internal stress of the layers during the swelling and exfoliation processes causes fracturing in the lateral direction. A similar size reduction with fracturing in the lateral direction was studied for the exfoliation of inorganic layered compounds,^18*b*^ even though such remarkable size reduction was not observed for organic layered materials in previous reports for different exfoliation methods.^25^

The exfoliated and dispersed PDA nanosheets were collected as restacked structures with addition of zinc ions (Zn^2+^) in the dispersion liquid. The negatively charged PDA nanosheets form precipitates with addition of the positively charged Zn^2+^. The collected precipitate showed the presence of nanoflakes of around 200 nm in lateral size (Fig. 3e). The lateral size is consistent with that of the nanosheets, as estimated by the AFM image (Fig. 3c and e). These results suggest that the exfoliated PDA nanosheets were generated in the dispersion liquid.

The hydrophilic PDA nanosheets in aqueous media were collected by evaporation of the water, although excess TBAOH as the delamination agent remained. The nanosheets showed absorption corresponding to a carboxylate group in the Fourier-transform infrared (FT-IR) spectrum (Fig. S3 in the ESI†), whereas the original layered PDA had a dimerized carboxy group in the hydrophilic interlayer space (Fig. 1). The change of the carboxy group is ascribed to the intercalation of a tetrabutylammonium cation (TBA^+^) in the hydrophilic interlayer space. The diffraction peaks originating from the layered structure were not observed in the XRD pattern of the collected nanosheets (profile (ii) in Fig. 2b). Although the diffraction peaks remained for the collected precipitates after dispersion for 10 min, these peaks disappeared after 24 h (Fig. S3 in the ESI†). These results imply that the exfoliation proceeds through the intercalation of TBA^+^ and subsequent osmotic swelling with water. The exfoliation process is similar to that of inorganic layered compounds.^2,16e,g^ If a similar layered structure with a hydrophilic interlayer space consisting of polar groups is designed and synthesized for organic materials (Fig. 1c), exfoliation into nanosheets can be achieved by the present approach.

### Exfoliation into hydrophobic PDA nanosheets

**Fig. 4:**
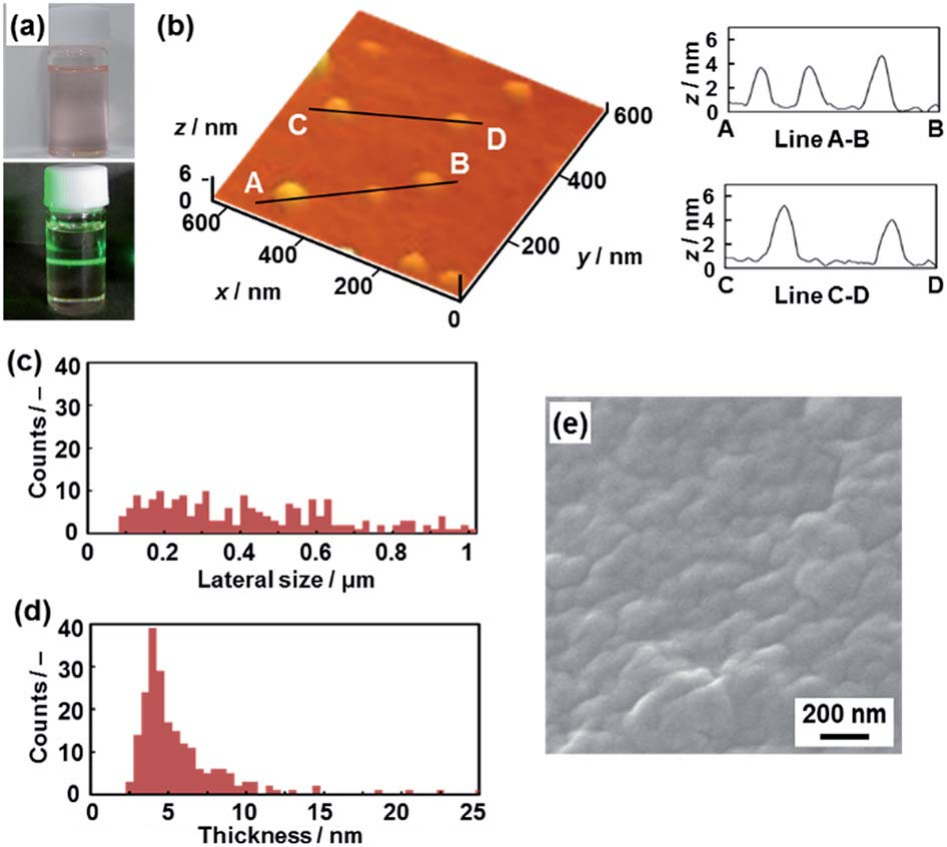
Hydrophobic PDA nanosheets. (a) Photographs of the toluene dispersion liquid (upper panel) with irradiation with laser light (lower panel). (b) AFM image of the hydrophobic nanosheets (left) and their height profiles (right). (c and d) Distribution of the lateral size (c) and the height (d). (e) Scanning electron microscope image of the restacked hydrophobic PDA nanosheets collected after evaporation of toluene. The additional AFM images are displayed in Fig. S5 in the ESI.†

The layered PDA has a hydrophobic interlayer space consisting of long-alkyl chains (Fig. 1). The hydrophobic PDA nanosheets were obtained by dispersion of the layered PDA in toluene (Fig. 4). The color of the PDA powder changed from blue to red after dispersion in toluene at 60 °C within 10 min. The color of the dispersion liquid became deep with an increase in the immersion time (Fig. S4 in the ESI†). After 10 days, the dispersed precipitate was collected by filtration and then redispersed in toluene at 60 °C. The remaining monomers and dissolved polymers were removed after the filtration. After a further 10 days, the transparent dispersion liquid with a red color was obtained after removal of bulk aggregates by centrifugation (upper panel in Fig. 4a). The light scattering implies the formation of nanoscale objects in the dispersion liquid (lower panel in Fig. 4a).

The nanosheets, which are approximately 5 nm in thickness, were observed in the AFM images (Fig. 4b and S5 in the ESI†). The distribution of the lateral size and thickness is summarized in the histograms (Fig. 4c and d). The distribution of the thickness with a peak around 5 nm is broadened compared with the hydrophilic nanosheets (Fig. 4c and d). The hydrophobic nanosheet consisted of a bilayered molecular arrangement (Fig. 1c). When the hydrophobic PDA nanosheets exhibiting long-alkyl chains on the surface are dispersed in toluene, the disordered arrangement of the alkyl chains causes the broadened distribution of the thickness. It is inferred that objects thicker than 6 nm correspond to nanosheets with a different layer number. The average lateral size of the nanosheets was 407 ± 247 nm. The lateral size was decreased from approximately 10 μm to 0.4 μm with fracturing in the lateral direction after dispersion in toluene (Fig. 2c and 4c). In this way, the hydrophobic PDA nanosheets are formed through exfoliation and fracturing in the cross-sectional and the lateral directions, respectively. The hydrophobic PDA nanosheets were collected by evaporation of toluene. The collected precipitates showed aggregates of the flake-like objects around 200 nm in lateral size (Fig. 4e). These results indicate the formation of the exfoliated nanosheets in the toluene media.

The hydrophobic PDA nanosheets were collected by evaporation of toluene. The interlayer carboxy group had not changed to the carboxylate one after dispersion in toluene (Fig. S6 in the ESI†). This result suggests that the intercalation of toluene and subsequent swelling proceeds in the hydrophobic interlayer space consisting of the alkyl chains (Fig. 1c). The diffraction peaks characteristic of the layered structure were weakened in the XRD pattern of the precipitates collected after dispersion for a day (Fig. S6 in the ESI†). The diffraction peaks completely disappeared for the collected precipitates after exfoliation for 3 weeks (profile (iii) in Fig. 2b). The hydrophobic PDA nanosheets were obtained by exfoliation through the intercalation of toluene in the hydrophobic interlayer space and subsequent swelling. If similar hydrophobic interlayer spaces consisting of interdigitated alkyl groups are designed in layered organic compounds, the present approach can induce exfoliation into the nanosheets.

### Photochemical properties of the PDA nanosheets

**Fig. 5:**
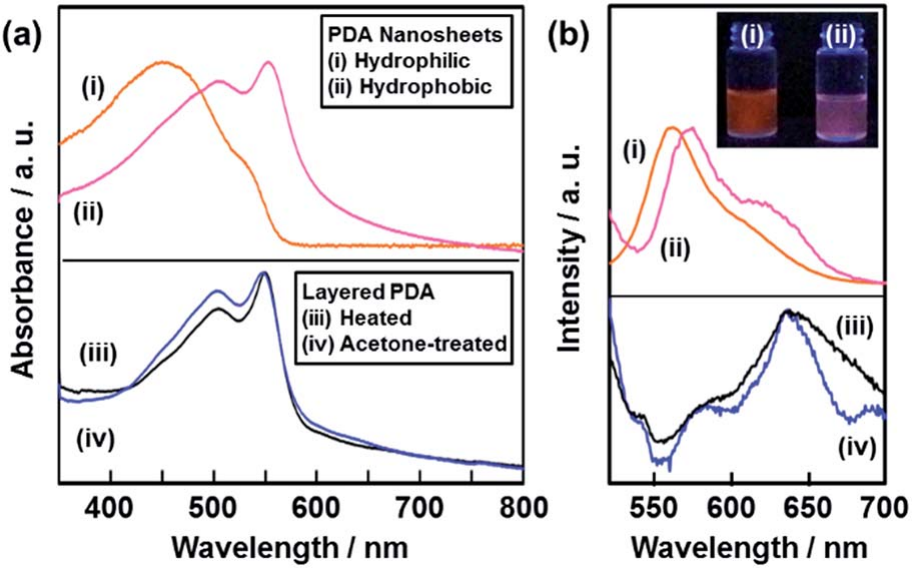
Photochemical properties of the PDA nanosheets and the reference samples. UV-Vis (a) and fluorescence (b) spectra of the hydrophilic nanosheets (i, orange) and the hydrophobic ones (ii, red), and the layered PDA with heating at 120 °C (iii, black) and immersion in acetone (iv, blue). The excitation wavelength of the fluorescence spectra was set at 488 nm. The inset of panel (b) shows the fluorescence images of the dispersion liquids containing the hydrophilic (i, the left) and hydrophobic (ii, the right) PDA nanosheets.

The resultant PDA nanosheets showed characteristic photochemical properties, such as absorption and fluorescence, originating from their flexible nature and diluted dispersion state (Fig. 5). The original layered PDA with a blue color showed broadened absorption peaks centered around 590 nm and 650 nm (Fig. S7 in the ESI†).^26*b*^ The spectrum was shifted to the shorter wavelength region around 450 nm and 550 nm for the hydrophilic and the hydrophobic PDA nanosheets, respectively (spectra (i) and (ii) in Fig. 5a). In general, the peak shift of the PDA derivatives with the color change from blue to red is observed upon application of external stimuli, such as temperature change, mechanical stress, and immersion in organic media.^27,28^ The external stimuli induce torsion of the PDA main chain with the motion of the alkyl side chain. The shortening of the conjugation length directs the color change from blue to red.^26,27^ When a powder of the precursor layered PDA was heated at 120 °C, the absorption peak was shifted to the shorter wavelength region at around 550 nm (spectrum (iii) in Fig. 5a).^26*b*^ A similar blueshift was observed for the powder of the layered PDA after immersion in acetone (spectrum (iv) in Fig. 5a). The hydrophilic PDA nanosheets showed a larger blueshift of the absorption peak to 450 nm (spectrum (i) in Fig. 5a). The large blueshift was not observed in previous studies, such as for the few-layered PDA derivatives prepared by the LB technique,^28^ the diluted state in polymer matrices,^29^ and the condensed crystalline state.^30^ The larger blueshift on the hydrophilic PDA nanosheets is ascribed to the shortening of the conjugation length with the flexible structure. Since the surface carboxylate groups of the hydrophilic PDA nanosheets are not anchored by cations in the aqueous dispersion liquid (Fig. 1c), the more flexible nature induces a large blueshift to 450 nm. In contrast, such flexibility is not achieved in the hydrophobic PDA nanosheets because the carboxy groups are anchored in the interlayer space (Fig. 1c).

The hydrophilic and hydrophobic PDA nanosheets showed a fluorescence peak at around 560 nm in the dispersion liquid (spectrum (i) and (ii) in Fig. 5b). In contrast, fluorescence peaks were observed at around 570 nm and 630 nm for the powder of the layered PDA after heating at 120 °C and immersion in acetone (spectra (iii) and (iv) in Fig. 5b). In general, no emission peak is observed for PDA with a blue-color state.^27,28^ In previous work, PDA-embedded organic materials, such as electrospun fibers and vesicles,^27c,d,29^ showed fluorescence peaks at 570 nm and 630 nm after application of external stimuli. The intensity ratio of the peaks at 570 nm and 630 nm depends on the incorporation states of PDA in the host materials.^27c,d,30a,31^ It is inferred that the diluted and condensed states of PDA provide fluorescence peaks at around 570 nm and 630 nm, respectively. The dominant fluorescence peak at around 570 nm was observed for the PDA-embedded polyester resin.^30*a*^ In the present work, the fluorescence peak at around 570 nm was dominant in the hydrophilic and hydrophobic PDA nanosheets in the dispersion liquid (spectra (i) and (ii) in Fig. 5b). The blue-shifted fluorescence peak of the PDA nanosheets is ascribed to the diluted state in the dispersion liquid. In general, photochemical properties of organic materials are tuned by design of molecules and their assembled states. In the present work, morphology control, such as formation of nanosheets through exfoliation, induces characteristic properties different from the bulk structures. These results imply that the ultrathin flexible nature of organic nanosheets has the potential for tuning of the properties. However, the characteristic physical properties originating from the nanosheet morphology were not found in the present work because the effective conjugation length of the PDA main chain determines the electronic properties at the molecular level. This is different to the case for inorganic nanosheet materials. Further study is required for exploration of the unprecedented properties of organic nanosheet materials.

### PDA nanosheets as a cross linker for a hydrogel

**Fig. 6:**
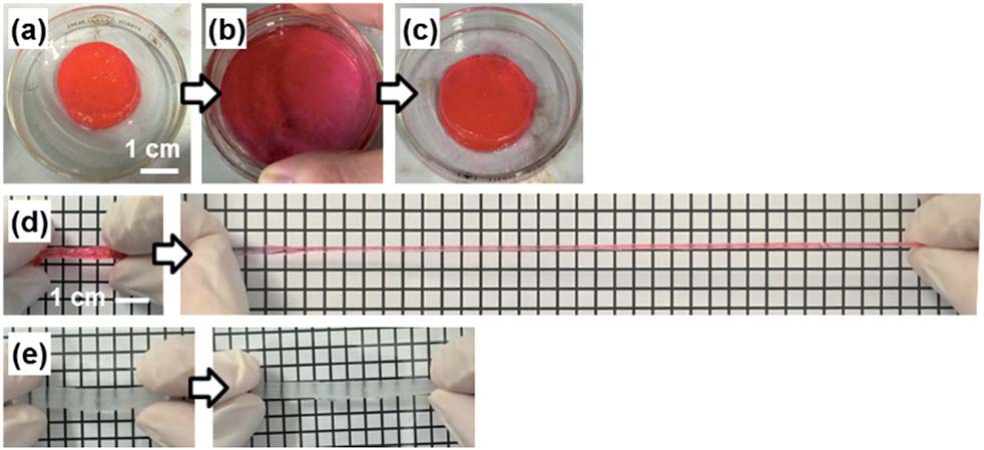
Photographs of the composite hydrogel consisting of pNIPAAm and the hydrophilic PDA nanosheets. (a) The original pNIPAAm/PDA composite gel. (b) The deformed gel after crushing. (c) The restored state. (d) The extended state of the pNIPAAm/PDA composite gel consisting of pNIPAAm and the hydrophilic PDA nanosheets. (e) The extended state of the conventional hydrogel consisting of pNIPAAm and MBAAm. The distance of the grid lines is 0.5 cm in panels (d) and (e).

The PDA nanosheet was used as a cross-linker for a hydrogel (Fig. 6). An aqueous dispersion of the hydrophilic PDA nanosheet formed a gel with addition of *N*-isopropylacrylamide (NIPAAm) as a monomer and potassium persulfate as an initiator (Fig. 6a). The detailed procedure is described in the ESI.† The dispersion liquid in the vessel changed to a gel state after 20 h (Fig. 6a). The composite gel of poly(NIPAAm) (pNIPAAm) and the PDA hydrophilic nanosheets was obtained without addition of an organic cross-linker. When the pNIPAAm/PDA composite gel was crushed and spread in a Petri dish, the deformed gel returned to its original shape within approximately 1 min (Fig. 6b and c). When the rod-like gel was stretched, the length was extended to be 8.9 times longer than its original state (Fig. 6d). In contrast, the gel cross-linked with a conventional organic linker, namely *N*,*N*′-methylenebisacrylamide (MBAAm), was broken upon extension by 1.6 times (Fig. 6e).

In previous work, a composite gel with similar elastic properties was prepared from an organic monomer and exfoliated inorganic clay as the cross-linker.^32^ When the nanosheets are included in the solution phase, the polymer chains are anchored on the surface of the nanosheets *via* the electrostatic interaction. Therefore, the stretchable polymer chains between the nanosheets contribute to the elastic properties. The results imply that exfoliated PDA nanosheets can be used as a building block for composite materials.

## Conclusions

Two exfoliation approaches were studied for syntheses of organic nanosheets. Layered PDA was exfoliated into hydrophilic and hydrophobic nanosheets in aqueous and nonpolar organic media, respectively. The intercalation of guests in the interlayer space induced swelling and subsequent exfoliation into PDA nanosheets consisting of a bilayer molecular arrangement around 5 nm in thickness. The resultant PDA nanosheets showed changes in their photochemical properties originating from their flexible nature and diluted state, different to the bulk material. In addition, the resultant PDA nanosheet was used as a building block for composite materials. The hydrogel of an organic polymer chain cross-linked with the PDA nanosheets showed unique stretching properties. If organic layered compounds with a similar hydrophilic and hydrophobic interlayer space are designed and synthesized, a variety of nanosheets can be obtained by the present exfoliation approaches. Organic nanosheets have the potential for the emergence of unprecedented properties that are different from the bulk structure. The present study contributes to exploring and expanding the potential of functional organic nanosheet materials.
