# Supplementary material for: Two exfoliation approaches for organic layered compounds: hydrophilic and hydrophobic polydiacetylene nanosheets
Source: Chem Sci. 2016 Aug 30;8(1):647–53. doi: 10.1039/c6sc03350d (PMC5297333; doi:10.1039/c6sc03350d)
Supplement: Supplementary file 1 [file SC-008-C6SC03350D-s001.pdf]

Electronic Supplementary Material (ESI) for Chemical Science.
This journal is © The Royal Society of Chemistry 2016

Electronic Supplementary Information (ESI)

Two exfoliation approaches for organic layered compounds: hydrophilic and hydrophobic polydiacetylene nanosheets

Yukiko Ishijima,[+] Mamoru Okaniwa,[+] Yuya Oaki,* Hiroaki Imai

Department of Applied Chemistry, Faculty of Science and Technology, Keio University, 3-14-1 Hiyoshi, Kohoku-ku, Yokohama 223-8522, Japan.

E-mail: oakiyuya@applc.keio.ac.jp

[+]These authors contributed equally to this work.

Contents

Experimental methods P. S2

Time-course observation of the exfoliation behavior in aqueous media (Fig. S1) P. S4

AFM images of the hydrophilic PDA nanosheets (Fig. S2) P. S5

FT-IR and XRD analyses of the hydrophilic PDA nanosheets (Fig. S3) P. S6

Time-course observation of the exfoliation behavior in nonaqueous media (Fig. S4) P. S8

AFM images of the hydrophobic PDA nanosheets (Fig. S5) P. S9

FT-IR analysis of the hydrophobic PDA nanosheets (Fig. S6) P. S10

UV-Vis spectrum of the precursor PDA (Fig. S7) P. S12

Experimental methods

Precursor organic layered compound: All the reagents were used without purification as purchased. The precursor layered PDA with blue color was obtained by the polymerization. A commercial powder of 10,12-pentacosadiynoic acid (PCDA, TCI, 97.0 %) as monomer was polymerized with irradiation of UV light and X-ray. The distribution of the lateral size and the thickness was analyzed by the images of scanning electron microscopy (SEM, Keyence, VE-9800). The sample for the SEM observation was coated by amorphous ultrathin osmium.

Synthesis and characterization of the hydrophilic PDA nanosheets: The precursor PDA powder, typically 10 mg, was dispersed in 20 cm^3 of an aqueous solution containing tetrabutlyammonium hydroxide (TBAOH, TCI, 40 % in water). The molar ratio of TBAOH to the PCDA monomer unit ($R_{TBAOH/PCDA}$) was adjusted to $R_{TBAOH/PCDA}$=4.0. The dispersion liquid was maintained under stirring at room temperature for 3 weeks. The lateral size and the thickness of the resultant PDA nanosheets were measured by atomic force microscopy (AFM, Hitachi, AFM 5000II). The dispersion liquid was centrifuged at 13500 rpm for 10 min to remove the bulk aggregates. However, the precipitate was not collected after the centrifugation. The aqueous dispersion of the PDA nanosheets was mixed with ethyl acetate (1/1 by volume) for 30 min to remove the remaining PCDA monomers. The separated aqueous phase was used as the dispersion liquid of the hydrophilic PDA nanosheets. The dispersion liquid was dropped on a silicon (Si) substrate for the AFM observation. A Si substrate was cleaned by immersion in the mixture of methanol and hydrochloric acid (1/1 by volume) and then the concentrated sulfuric acid for 30 min. After washing by purified water, the cleaned Si substrate was immersed in 2.0 g dm^{-3} of polyethyleneimine (Acros Organics, PEI, M_w=6.0×10^4, 50 wt.-% aqueous solution) aqueous solution for 15 min to obtain the positively charged surface. Then, the excess amount of PEI was removed by washing with purified water. The PEI-coated substrate was immersed in the aqueous dispersion liquid of the PDA nanosheets. The excess PDA precipitates

were removed by washing with purified water. The photochemical properties of the PDA nanosheets in the dispersion liquid were analyzed by UV-Vis spectrophotometer (Jasco, V-670) and spectrofluorophotometer (Jasco, FP-6500). Water in the dispersion liquid was evaporated to measure the X-ray diffraction (XRD, Bruker, D8-Advance) pattern and Fourier-transform infrared (FT-IR, Jasco, FT/IR-4200) spectrum.

Synthesis and characterization of the hydrophobic PDA nanosheets: The precursor PDA powder, typically 10 mg, was immersed in 20 cm^3 of toluene. The dispersion liquid was maintained under stirring at 60 ˚C for 10 days. The precipitate was collected by filtration to remove the remaining PCDA monomer dissolved in toluene. The filtrated precipitate was then dispersed in toluene under stirring at 60 ˚C for the further 10 days. The bulky aggregate was removed by centrifugation at 6600 rpm for 10 min. The resultant PDA dispersion liquid was used for the characterization. The lateral size and the thickness of the hydrophobic PDA nanosheets were measured by AFM. For AFM observation, the resultant dispersion liquid was diluted and then dropped on a cleaned silicon substrate put on a temperature-controlled sample stage heated at 120 ˚C. For the XRD and FT-IR analyses, the exfoliated nanosheets were collected by evaporation of toluene from the dispersion liquid. The UV-Vis and fluorescent spectra were obtained from the dispersion liquid.

Time-course observation of the exfoliation behavior in aqueous media

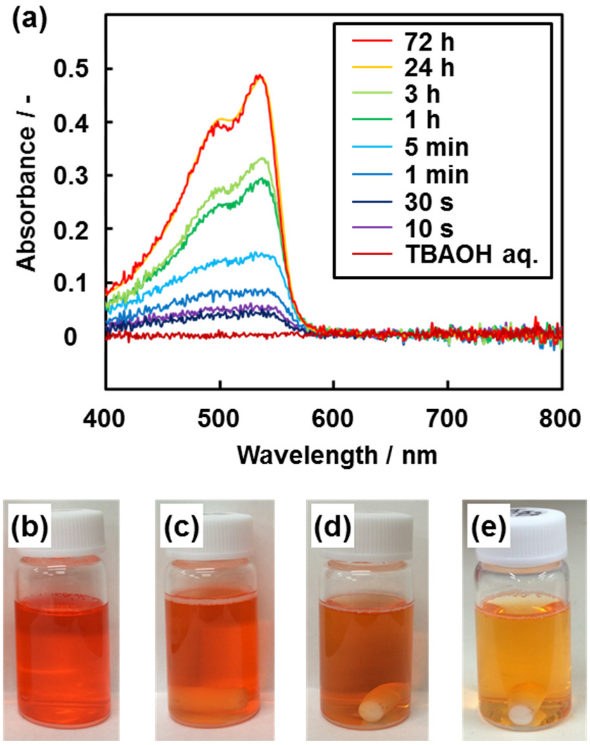

Fig. S1. Time-course observation of the aqueous dispersion liquid of the layered PDA. (a) UV-Vis spectra of the dispersion liquids in the initial stage of the exfoliation. (b–e) Photographs of the dispersion liquid after 3 days (b), 1 week (c), 2 weeks (d), and 3 weeks (e).

In the initial stage, the absorbance of the dispersion liquid was increased with an increase in the time after the dispersion in an aqueous solution containing TBAOH (Fig. S1a). The results suggest that the exfoliated PDA nanosheets were formed in the aqueous medium. The further increase in the immersion time induces the color change from red to orange (Fig. S1b–e). Since the fracture in the lateral direction proceeds in the long-term immersion, the more flexible nature and the smaller lateral size induce the color change originating from the shortening of the conjugation length.

AFM images of the hydrophilic PDA nanosheets

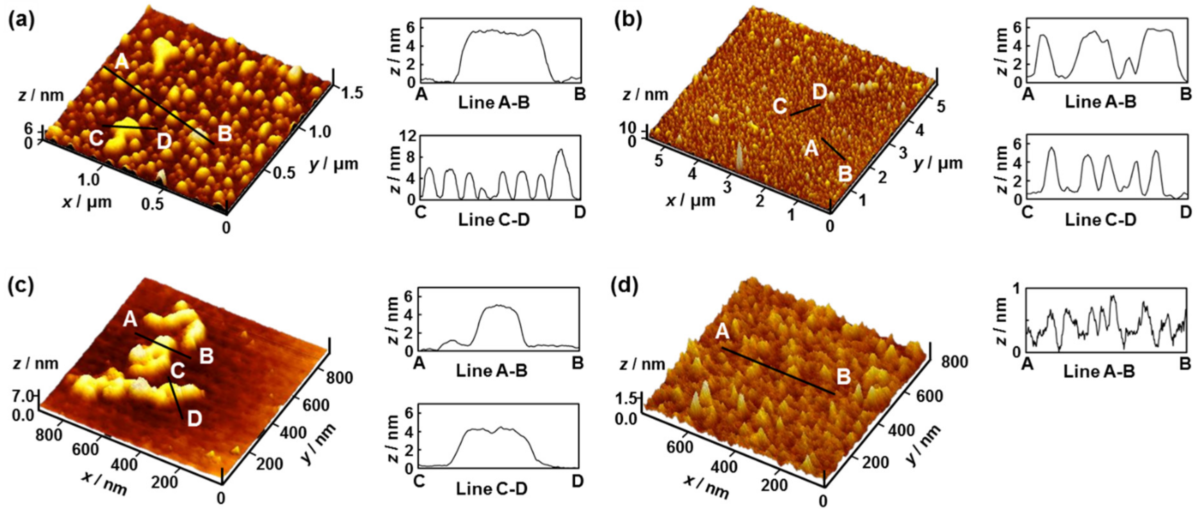

Fig. S2. Additional AFM images of the hydrophilic PDA nanosheets. (a–c) AFM images of the PDA nanosheets obtained after dispersion for 3 weeks (a,b) and 3 days (c). (d) AFM image of the PEI-coated silicon substrate before the casting of the PDA nanosheets.

In addition to the AFM image in Fig. 3b, the hydrophilic PDA nanosheets were obtained throughout the substrate (Fig. S2a,b). The PDA nanosheets were observed after 3 days of the dispersion (Fig. S2c). The PEI-coated silicon substrate was immersed in the aqueous dispersion liquid of the PDA nanosheets. The negatively-charged PDA nanosheets were adsorbed on the surface via electrostatic interaction. The PEI-coated silicon substrate showed no platy objects higher than 1.0 nm in thickness (Fig. S2d). Therefore, the platy objects around 5 nm in thickness are regarded as the resultant PDA nanosheets.

FT-IR and XRD analyses of the hydrophilic PDA nanosheets

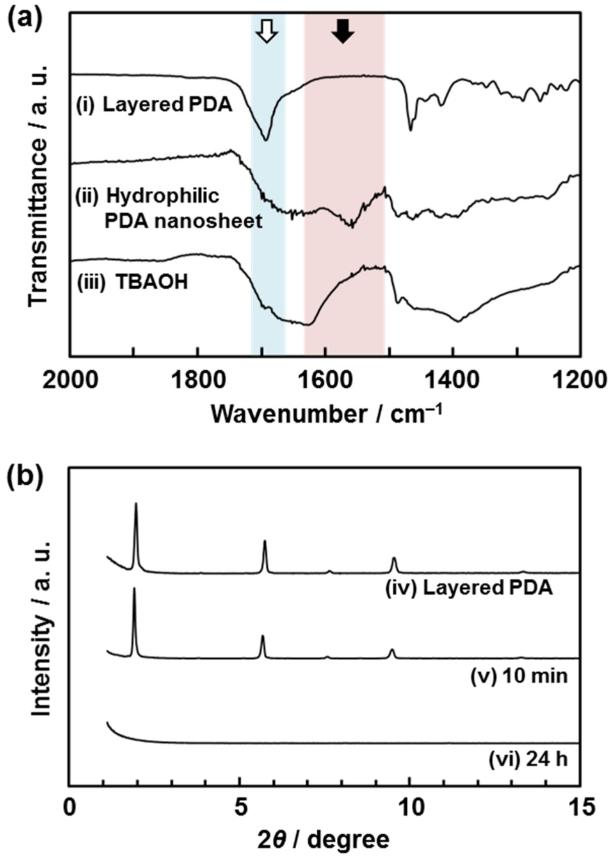

Fig. S3. FT-IR spectra of the layered PDA (i), the hydrophilic PDA nanosheets after the exfoliation (ii), the TBAOH aqueous solution after evaporation of water (iii). XRD patterns of the original layered PDA (iv), the collected precipitates after the dispersion in the TBAOH aqueous solution after 10 min (v) and 24 h (vi).

The dimerized carboxy groups were changed to carboxylate ones after intercalation of TBA^+ in the hydrophilic interlayer space consisting of the carboxy groups. The stretching vibration of C=O bond in the intramolecular dimerized carboxy group was only observed on the precursor layered PDA around 1700 cm^{-1} (the white arrow with the blue background in Fig. S3a). The dispersion liquid of the exfoliated PDA nanosheets was dried with evaporation of water. The PDA nanosheet sample showed the absorption corresponding to the carboxylate group around

1550 cm^{-1} (the black arrow with the red background in Fig. S3a), whereas the absorbance around 1700 cm^{-1} originating from the dimerized carboxy group was decreased. The results indicate that the intercalation of TBA^+ proceeds in the hydrophilic interlayer space.

When the precursor layered PDA was dispersed in the TBAOH aqueous solution, the peaks corresponding to the layered structure were kept after 10 min (the profiles (iv) and (v) in Fig. S3b). However, the characteristic peaks disappeared after 24 h (the profile (vi) in Fig. S3b). These results indicate that the exfoliation with disappearance of the periodic layered structure proceeds after 10 min.

Time-course observation of the exfoliation behavior in nonaqueous media

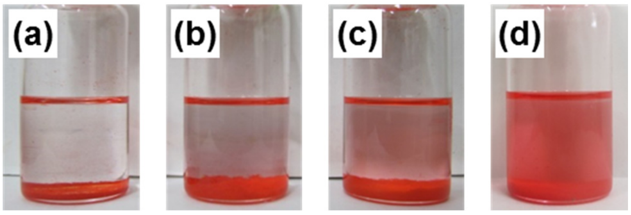

Fig. S4. Photographs of the toluene dispersion liquid at 5 days (a), 1 week (b), 2 weeks (c), and 3 weeks (d).

The dispersion liquid was colored to red with an increase in the immersion time. The bulk precipitate remained on the bottom of the toluene dispersion liquid (Fig. S4), whereas the bulk precipitate was not observed in the aqueous dispersion liquid containing TBAOH (Fig. S1b–e). The bulk precipitate was removed by centrifugation to obtain the dispersion liquid of the hydrophobic PDA nanosheets (Fig. 3e).

AFM images of the hydrophobic PDA nanosheets

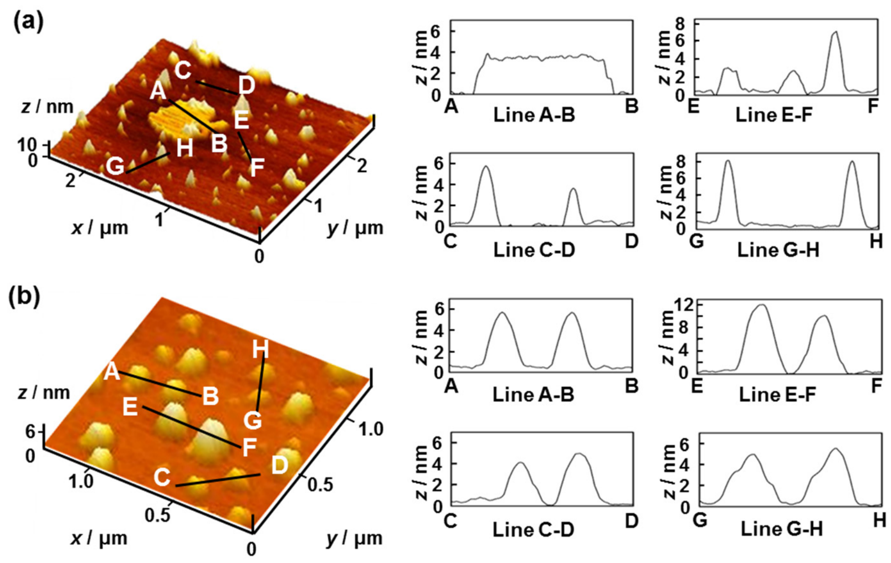

Fig. S5. Additional AFM images of the hydrophobic PDA nanosheets obtained after dispersion for 3 weeks.

In addition to the AFM image in Fig. 3f, the hydrophobic PDA nanosheets were observed on a cleaned silicon substrate (Fig. S5). The platy objects around 5 nm in thickness were observed throughout the substrate. In addition, the thicker objects than ca. 6 nm were observed on the substrate. Since the hydrophobic PDA nanosheets are prepared in toluene, the dissolution of the remaining monomer and the redeposition respectively cause the broadening of the thickness distribution. The hydrophobic PDA nanosheets exhibiting the long-alkyl chain were dispersed in toluene. Therefore, the disordered arrangement of the alkyl chains causes the broadened distribution of the thickness.

FT-IR analysis of the hydrophobic PDA nanosheets

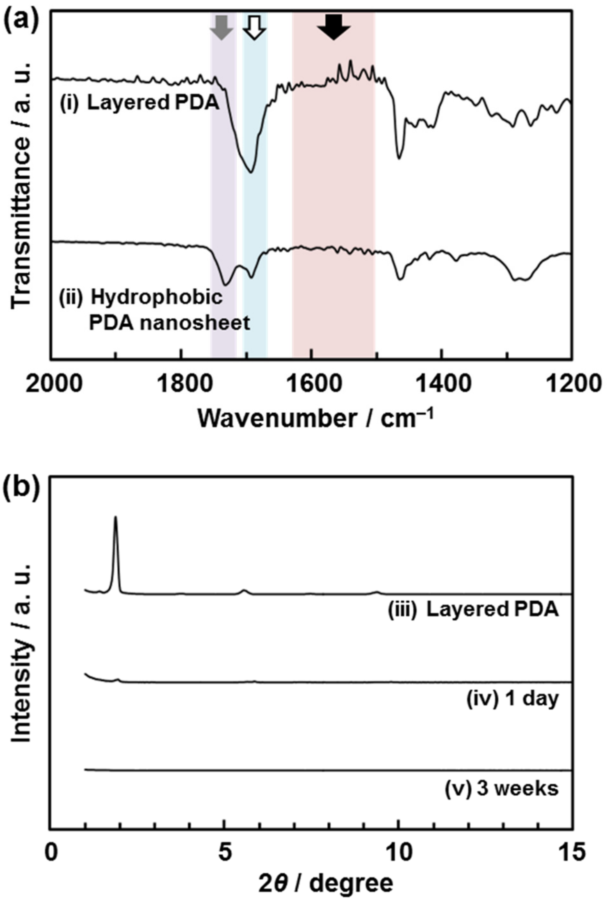

Fig. S6. FT-IR spectra of the layered PDA (i), the hydrophobic PDA nanosheets after the exfoliation and evaporation of toluene (ii). XRD patterns of the layered PDA (iii), the collected precipitates after the dispersion in toluene after 1 day (iv) and 3 weeks (v).

While the absorbance corresponding to the dimerized carboxy group around 1700 cm^{-1} was slightly decreased with an increase in the immersion time (the white arrow with blue background in Fig. S6a), the absorbance of the monomeric carboxy group around 1760 cm^{-1} appeared (the gray arrow with the purple background in Fig. S6a). Nevertheless, the absorbance originating from the carboxylate group around 1550 cm^{-1} was not observed after the immersion

in toluene for 3 weeks (the black arrow with red background in Fig. S6a). The results suggest that toluene molecule was intercalated not in the hydrophilic interlayer space consisting of the dimerized carboxy groups but in the hydrophobic one consisting of the long alkyl chains.

When the precursor layered PDA was dispersed in toluene, the peaks corresponding to the layered structure were drastically weakened after a day (the profiles (iii) and (iv) in Fig. S6b). The characteristic peaks completely disappeared after 3 weeks (the profile (v) in Fig. S6b).

UV-Vis spectrum of the precursor PDA

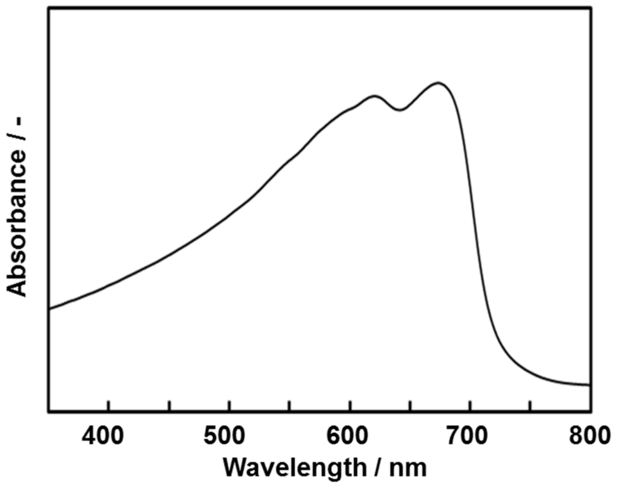

Fig. S7. UV-Vis spectrum of the precursor PDA with the blue color state before application of external stimuli.

The original layered PDA with blue color showed the broadened absorption peak centered around 590 nm and 650 nm.[26b]
